# A Real-World Study of the Association between a Brief Group Psychoeducation and the Course of Bipolar Disorder

**DOI:** 10.3390/ijerph18095019

**Published:** 2021-05-10

**Authors:** Elisabet Casellas, Beatriz Raventós, Marina Piñeiro-Ríos, Helena Navarro-Martínez, Maite Castillón-Espezel, Maria J. Portella, Ana Martín-Blanco

**Affiliations:** 1 Sant Pau Biomedical Research Institute (IIB-Sant Pau), Department of Psychiatry, Hospital de la Santa Creu i Sant Pau, 08041 Barcelona, Spain; ecasellas@santpau.cat (E.C.); mportella@santpau.cat (M.J.P.); 2 Centre de Salut Mental D’adults (CSMA) de Sarria-Sant Gervasi, Associació Centre D’higiene Mental Les Corts, Grup CHM Salut Mental, 08029 Barcelona, Spain; beatriz.raventos@chmcorts.com; 3 Department of Psychiatry, Hospital Universitari Mútua de Terrassa, 08221 Barcelona, Spain; mpineiro@mutuaterrassa.cat; 4 Institut de Neuropsiquiatria I Addiccions (INAD), Parc de Salut Mar, 08003 Barcelona, Spain; Helena.nama@gmail.com; 5 Department of Psychiatry, Consorci Sanitari de Mataró, 08304 Mataró, Spain; mcastillon@santpau.cat; 6 Centro de Investigación Biomédica en Red de Salud Mental (CIBERSAM), 28029 Madrid, Spain

**Keywords:** bipolar disorder, group psychoeducation, disease progression, recurrence, treatment adherence, insight

## Abstract

Although pharmacotherapy is considered the first-line treatment for bipolar disorders (BD), adjunctive psychoeducation has proven its effectiveness in improving self-management of the disease and reducing relapse rates. Few studies have evaluated the effect of brief group psychoeducation on pragmatic variables, such as the number of hospitalizations. The aim of the present study was to assess the mid-term effect of a four-session group psychoeducation on course-related variables in BD. Thirty-two individuals with BD were included in the study. Sixteen were exposed to psychoeducation and were matched to sixteen nonexposed individuals who received their usual treatment. Both groups were compared on insight, treatment adherence, change in the number of hospitalizations and visits to the emergency services, occurrence rate after intervention, and time to the first psychiatric hospitalization and the first urgent attendance. There was a significant reduction in the mean number of hospitalizations and urgent attendances in the exposed group in comparison to the nonexposed group. The first urgent attendance was significantly sooner in the nonexposed cohort. There were no differences between groups in any of the other variables. This intervention has shown benefits for pragmatic variables of the disease course and may be a feasible and cost-effective intervention to routinely implement in the management of BD.

## 1. Introduction

Bipolar disorder (BD) is a chronic and recurrent illness characterized by mood oscillation episodes associated with a reduced quality of life [1,2] and a high social and economic burden [3,4]. Although pharmacotherapy is the first-line treatment [5], several studies point to nonadherence rates of approximately 50% [6], which increases the recurrence risk and worsens the course of the disorder [7]. In this sense, adjunctive psychological interventions—especially those with a psychoeducation component—have proven to be effective in reducing relapse rates and improving self-management of the disease [8,9,10]. Specifically, according to some recent systematic reviews [11,12], adjunctive psychoeducation has shown effects on relapse prevention, medication adherence, and short-term knowledge about medication (but not on mood symptoms, quality of life, or psychosocial functioning). Moreover, a randomized controlled study comparing group psychoeducation with nonstructured group intervention during a 5-year follow-up has found that the former also increases time to recurrence [13], reduces hospitalization length [14], and decreases medical costs [15].

Most studies on group psychoeducation perform long therapies, usually up to 21 sessions, while shorter ones would be even more cost-effective. In this line, some authors have proposed brief psychoeducation interventions, ranging from four sessions [16,17] to eight sessions [18,19]. These latter interventions have been shown to improve bipolar symptomatology [18,20], internalized stigmatization [21], knowledge of the disorder [22], relapse rates [18,19], quality of life [19], psychosocial functioning [17,18], and medication adherence [23]. However, there are few studies [18,19] evaluating their effect using more pragmatic variables, such as the number of hospitalizations or time to recurrence, which provide objective measures of effectiveness. Besides, these previous studies have not considered the role of insight, which has been suggested to improve treatment adherence, and in consequence, disease prognosis [24]. Therefore, it is necessary to gain knowledge on the efficacy of brief structured group programs to facilitate their implementation in public health system contexts.

To this purpose, the present study aimed at assessing the mid-term effect of a brief group psychoeducation program (with both information and coping skills components) on the objective measurement of course-related variables in a sample of bipolar patients. The implementation of feasible and cost-effective interventions is to be investigated so as to provide real-world data, which should guide public health decisions.

## 2. Materials and Methods

### 2.1. Patients and Study Design

This was a retrospective cohort study conducted in outpatient facilities of the Department of Psychiatry of the Hospital de la Santa Creu i Sant Pau (Barcelona, Spain). All participants were adults (age ≥ 18 years old) meeting criteria for bipolar I disorder according to the Diagnostic and Statistical Manual of Mental Disorders (DSM-IV-TR). The total sample included 32 subjects, 16 of whom had received the exposition.

In this study, exposition refers to having participated to brief psychoeducation groups consecutively held in the unit, from September 2015 to April 2017. Attendance to these groups is open to all outpatients. These structured groups consist of four weekly sessions of 90 min each, imparted by two clinical psychologists. We designed the intervention based on the Spanish version of the Psychoeducation Manual for Bipolar Disorders by Drs. Colom & Vieta [25], as it has amply proven its effectiveness in clinical trials [14,26]. We dedicated a session to each of the different modules that they propose, combining the modules of avoidance of substance use and self-care measures and stress management in one session. The contents of the sessions are divided into definition and clinical manifestations of the disorder (session 1); etiology and treatment (session 2); avoidance of substance use, self-care measures, and behavioral strategies (session 3); and relapse prevention and personal projects (session 4). During this intervention, the subjects are allowed to maintain their usual psychiatric controls and pharmacological treatment. The patients are commonly referred by their regular psychiatrist, and the only exclusion criterion is having an intellectual disability to understand the contents. For the purpose of the current work, 23 patients fulfilling inclusion criteria to participate started the intervention, and the final sample for the present analysis consisted in 16 individuals who attended at least three out of the four sessions. Excluded exposed patients did not differ from the rest of the participants in terms of basal sociodemographic and clinical characteristics (see Supplementary Material).

The nonexposed cohort was composed of 16 outpatients also affected by bipolar I disorder, whose psychiatrist did not refer them to psychoeducation intervention (specific reasons could not be gathered). To avoid differences between exposed and nonexposed individuals, both groups were matched by educational level, years until bipolar diagnosis, basal level of insight, and basal level of medication adherence. The educational level was considered because this factor may influence the results of psychosocial interventions. The reason for pairing by basal level of insight and treatment adherence was to prevent the outcomes from being biased by initial differences between cohorts. For each subject in the exposed group, his/her matched pair was randomly selected among those outpatients meeting the matching characteristics. These 16 nonexposed subjects had been receiving their usual pharmacological treatment and psychiatric controls while their exposed pairs attended the group psychoeducation. The study time points coincided in both groups.

The following outcomes were compared between the exposed and the nonexposed cohorts:

Insight improvement: a comparison of the insight level at baseline and a year after the intervention, to determine if there had been an improvement or not. The insight level at each point was classified as poor, partial, or good, according to the patients’ psychiatrists’ clinical impression. This information was obtained from the patients’ routine medical records; if it was missing, we directly asked the patients’ psychiatrists to classify their insight into poor, partial, or good. Improvement was defined as changing from poor at baseline to partial or good after a year, or from partial to good.

Treatment adherence improvement: a comparison of the treatment adherence at baseline and a year after the intervention, to determine if there had been an improvement or not. The treatment adherence at each point was evaluated by checking the registers of the plasmatic levels of the drugs, and was classified into poor, partial, or good. Improvement was defined as changing from poor at baseline to partial or good after a year, or from partial to good.

Change in the number of psychiatric hospitalizations: the number of hospitalizations during the year after the intervention was compared to the number of hospitalizations during the previous year.

Change in the number of visits to the psychiatric emergency services: the number of emergency consultations for depressive or hypomanic/manic symptoms during the year after the intervention was compared to the number of consultations during the previous year.

Occurrence rate of psychiatric hospitalizations and visits to the psychiatric emergency services after the end of the group psychoeducation.

Time to the first psychiatric hospitalization and the first visit to the psychiatric emergency services after the end of the group psychoeducation.

Our hospital belongs to the public national health system, which is free and universal. Patients can visit the psychiatric emergency service whenever they need to, and the decision to carry out a psychiatric hospitalization is made by the psychiatrist in the emergency room according to the severity of symptoms. It can also be taken by the patient’s regular psychiatrist.

All the information was obtained from patients’ medical records and/or by asking their regular psychiatrists in order to avoid missing data. The study was approved by the Clinical Research Ethics Committee at the Hospital de la Santa Creu i Sant Pau, and it followed the principles outlined in the Helsinki Declaration of 1975.

### 2.2. Statistical Analysis

Data were analyzed using the StataIC 13.1 (StataCorp, College Station, TX, USA).

Descriptive statistics were performed to describe the sociodemographic and clinical profile of both cohorts (exposed and nonexposed) at baseline. Quantitative variables were compared using a Student’s *t*-test for independent samples (since they followed a normal distribution—assessed by means of the Shapiro–Wilk test for normal data—and had equal variances—assessed by means of the Levene’s test). For categorical variables, chi-square tests were performed (or Fisher’s exact tests if the expected frequencies were <5).

Improvements in insight and treatment adherence were analyzed by comparing the proportions for matched data. Their 95% confidence intervals (95% CI) were also calculated. Changes in the number of hospitalizations were analyzed by comparing the differences of means (mean number of hospitalizations one-year post-group—mean number of hospitalizations the previous year) between both cohorts. Since this variable followed the assumptions of normality and homoscedasticity, a Student’s *t*-test for independent samples was performed. The same method was conducted for the number of visits to the psychiatric emergency services, but, in this case, a two-sample Wilcoxon rank-sum test was performed, since this variable did not follow a normal distribution.

To determine if there were differences between both cohorts in the occurrence rate of hospitalizations, a Cox’s regression model for recurrent events (counting the process model of Andersen–Gill [27]) was used. The last observation for the subjects who did not need hospitalization (right censored observations), independently of their date of inclusion in the study, was 13 April 2018 (a year after the last group used for the current analysis). Afterwards, this analysis was repeated while including the presence of psychotic features as a confounding factor (rapid-cycling was dismissed due to its low frequency in the sample). The same analyses were performed for visits to psychiatric emergency services. Finally, survival analyses were conducted to detect possible differences between cohorts in the elapsed time between the end of the intervention and the first hospitalization. To compare both cohorts, the generalized Wilcoxon method (Breslow test) was used. Moreover, a Cox regression was performed to obtain hazard ratios. The same procedure was followed to determine if there were differences between cohorts in the elapsed time between their inclusion in the study and their first emergency attendance. All hypotheses were tested with a two-sided significance level of 0.05.

## 3. Results

### 3.1. Demographic and Clinical Characteristics of the Sample

Table 1 summarizes the demographic and clinical profile of the sample. As shown, there were no statistical differences between the groups in terms of age, sex, duration of illness, educational level, basal level of insight, or treatment adherence.

### 3.2. Study of the Association between the Intervention and Insight and Treatment Adherence Improvement

There were no statistically significant differences between the groups, either in terms of insight improvement (RR = 1.500; 95% CI = 0.251–8.977) or in terms of treatment adherence improvement (RR = 1.000; 95% CI = 0.141–7.099).

### 3.3. Study of the Association between the Intervention and the Number of Hospitalizations and Visits to Emergency Services

**Figure 1:**
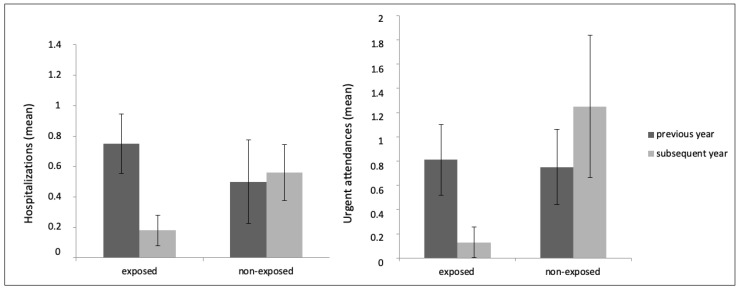
Mean number of psychiatric hospitalizations (**left**) and urgent attendances (**right**) in the intervention and the nonexposed cohorts one year before and one year after the group psychoeducation.

As shown in Figure 1 (left), there was a reduction in the mean number of psychiatric hospitalizations in the intervention group and an increase in the nonexposed group. The difference between the mean differences of both cohorts was statistically significant (*p* = 0.018). There was also a decrease in the mean number of visits to psychiatric emergency services in the exposed group and an increase in the nonexposed one (see right side of Figure 1), and the difference between the mean differences of cohorts was statistically significant (*p* = 0.029).

### 3.4. Study of the Association between the Intervention and the Rates of Hospitalizations and Visits to Emergency Services

Most of the psychiatric hospitalizations and visits to psychiatric emergency services were due to manic episodes. Specifically, manic symptoms were the cause of 100% of the hospitalizations and 40% of the emergency attendances in the exposed cohort, and of 78.6% of the hospitalizations and 75% of the emergency attendances in the nonexposed cohort. When analyzing the raw data, there were no differences between the groups either in terms of the occurrence rate of hospitalizations (HR = 0.44; 95% CI = 0.17–1.14) or in terms of the occurrence rate of urgent attendances (HR = 0.25; 95% CI = 0.05–1.16). However, when controlling for the presence or absence of psychotic features, the rate of hospitalization was significantly lower for the exposed group (HR = 0.42; 95% CI = 0.18–0.96). This means that the estimated risk of hospitalization for those subjects that were nonexposed to the brief psychoeducation intervention was 2.39 times higher than the risk for the exposed subjects (95% CI = 1.05–5.44). There were no differences between the groups in terms of urgent attendances (HR = 0.25; 95% CI = 0.06–1.13). A summary of these results is exposed in Table 2.

### 3.5. Study of the Association between the Intervention and the Lapsed Time until the First Hospitalization and the First Visit to the Emergency Services

Even though the hazard ratios of the first psychiatric hospitalization (HR_H_) and first psychiatric urgent attendance (HR_U_) were higher in the nonexposed cohort, these differences were not statistically significant (HR_H_ = 2.16; 95% CI = 0.70–6.61; HR_U_ = 3.61; 95% CI = 0.73–17.89).

**Figure 2:**
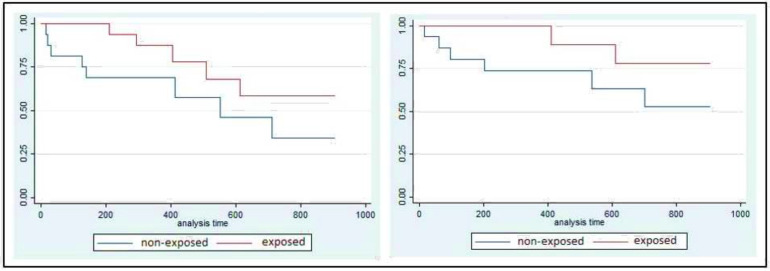
Survival curves representing the effect of the brief group psychoeducation on the elapsed time (days) until the first psychiatric hospitalization (**left**) and the first psychiatric urgent attendance (**right**). These differences in favor of the exposed cohort were only statistically significant for the urgent attendances.

As represented in Figure 2, the participants in the nonexposed cohort needed hospitalization or urgent assistance sooner than the participants in the intervention cohort. However, differences between groups were only significant for the first visit to emergency services (*p* = 0.046).

## 4. Discussion

Our results show that this brief group psychoeducation for subjects with bipolar I disorder may be effective in reducing the number of psychiatric hospitalizations and urgent attendances in the following months, as well as in increasing the time to the first urgent attendance. The design of the study does not allow one to draw robust conclusions, but these promising preliminary results encourage one to perform experimental studies to properly evaluate its efficacy.

The effectiveness of adjunctive group psychoeducation in improving the course of bipolar disorder has been extensively proven [14], but the length of these interventions can last over six months. This circumstance has driven some authors to design shorter interventions, but more evidence is needed to confirm their effectiveness and impact on objective measurements in the real world. Additionally, shorter group psychoeducation programs might be even more cost-effective, facilitating implementation in clinical settings. In this sense, our results provide more evidence to the scarce available literature, showing an association between the brief group psychoeducation and an improvement of the course of the disorder along the first year after the intervention. Indeed, the exposed cohort needed fewer hospitalizations, maintained clinical stability for longer, and attended emergency services less frequently along the follow-up. Moreover, when controlling for the presence of psychotic features, the psychoeducation program was associated with a decrease in the occurrence rate of hospitalizations but not of urgent attendances. This may suggest that the effect of this intervention is strong at the beginning but decreases over time. If this lessening is confirmed, efforts should be directed to periodically repeat the group psychoeducation or, at least, to offer reminder sessions of the contents either face-to-face or on the internet (i.e., website or mobile app). In fact, there is growing interest in internet-based interventions, and some psychoeducation programs for bipolar disorder are now being tested [28,29]. Offering at first a therapist-facilitated psychoeducation instead of directly receiving a self-administered psychoeducation would be better for the disorder’s course, as supported by some evidence [22]. Even though this psychoeducation program was not designed to prevent a specific sort of mood episode (i.e., manic, depressive, or mixed), it would be interesting to study if there is an association between this intervention and a reduction in the incidence of a specific polarity. However, the low occurrence of depressive and mixed episodes in our sample along the follow-up do not allow us to conduct these analyses.

Contrary to our expectation, this brief intervention was not able to improve either the insight or the pharmacological adherence of our patients. One reason may be that many subjects in both cohorts had good basal levels of insight and treatment adherence (see Table S1), and there might therefore not be room for improvement. However, there are other feasible explanations. For instance, in this study the insight was classified into three categories, while this is a complex and multidimensional construct [30]. Using a questionnaire based on multidimensional approaches (e.g., Insight Scale for Affective Disorders [31]) would have been more appropriate for detecting changes due to psychoeducation. With regard to treatment adherence, a relatively recent systematic review concludes that psychoeducation improves treatment adherence [14]; nevertheless, a number of subsequent studies do not support this assumption [18,32]. Moreover, some studies assessed medication adherence using questionnaires instead of more reliable measures such as plasmatic levels. Therefore, this issue requires further attention. What seems clear is that administering a specific intervention targeting medication adherence might be effective [33,34], although focusing only on this aspect might be detrimental to training self-managing and coping skills, abilities that may favorably affect the course of the disorder. In fact, the beneficial effects of the brief group psychoeducation may have been a consequence of the acquisition of such abilities, inasmuch as insight or treatment compliance had not changed. Therefore, this could be an explanatory hypothesis for the association between the psychoeducation intervention and the decrease in the number of psychiatric hospitalizations and visits to the psychiatric emergency services, although no changes in either insight or treatment adherence were observed. The acquisition of coping skills and self-care strategies may explain the better symptom-managing of the exposed group.

Despite its promising results, this is a pilot study with limitations, such as a small sample size and limited follow-up period. To validate these results, a randomized controlled trial should be conducted with a larger sample, a more accurate assessment of insight, and a longer follow-up period. This latter aspect would enable one to test whether treatment effects dissipate over time, and whether or not it may require additional prompting after the conclusion of the intervention. Given the observational nature of the present study, it was not possible to gather specific reasons for the nonreferral to the group intervention of the nonexposed cohort. This is a controversial issue for interpreting the results, but in any case special efforts in matching the two groups were made so as to have comparable individuals. Another limitation was that this intervention was administered in patients with bipolar I disorder, so these results cannot be generalized to patients with other types of the disorder. Besides these weaknesses, this study provided clinically relevant information since it was carried out in the context of the usual clinical practice, reflecting the conditions and resources available in the Spanish health system. The implementation of common strategies such as brief group psychoeducation, as well as monitoring and evaluating them, can be useful as the results allow one to guide evidence-based therapeutic decisions for BD patients.

Studying the impact of these interventions, specifically on objective indicators, would motivate their inclusion in the routine treatment of people with BD beyond pharmacological intervention and symptom management.

## 5. Conclusions

In conclusion, this brief four-session group psychoeducation may be a feasible and cost-effective intervention to routinely implement in the management of bipolar I disorder. The benefits of this program on pragmatic variables of the disease course provide useful information favoring public health stakeholders’ decisions regarding cost-effective interventions.

## Figures and Tables

**Table 1:** Demographic and clinical characteristics of the sample. The exposed cohort comprised individuals who attended one of the three groups of psychoeducation included in the analyses. *: Fisher’s exact test.

	Exposed Cohort	Non-Exposed Cohort	Statistical Test	*p*-Value
Age [mean (SD)]	37.19 (6.91)	40.38 (9.75)	t = 1.0672	0.2944
Sex, women [N (%)]	9 (56.25%)	9 (56.25%)	chi^2^ = 0.0000	1.0000
Duration of illness, years [M (SD)]	8.31 (8.87)	10.5 (10.53)	t = 0.6355	0.5299
Educational level [N (%)]			chi^2^ = 0.5818	0.446
Primary studies	0 (0%)	0 (0%)		
Secondary studies	6 (37.50%)	4 (25.00%)		
Superior studies	10 (62.50%)	12 (75.00%)		
Basal insight level [N (%)]			chi^2^ = 1.6769	0.504 *
Poor	1 (6.25%)	3 (18.75%)		
Partial	6 (37.50%)	7 (43.75%)		
Good	9 (56.25%)	6 (37.50%)		
Basal treatment adherence level [N (%)]			chi^2^ = 0.5333	1.000 *
Poor	2 (12.50%)	3 (18.75%)		
Partial	2 (12.50%)	1 (6.25%)		
Good	12 (75.00%)	12 (75.00%)		
Rapid-cycling [N (%)]	1 (6.25%)	1 (6.25%)	chi^2^ = 0.000	1.000 *
Psychotic features [N (%)]	11 (68.75%)	10 (62.50%)	chi^2^ = 0.1385	0.710
Basal pharmacological treatment [N (%)]				
Mood stabilizers	15 (93.75%)	16 (100%)	chi^2^ = 1.0323	1.000 *
Antipsychotics	9 (56.25%)	9 (56.25%)	chi^2^ = 0.0000	1.000 *
Antidepressants	3 (18.75%)	3 (18.75%)	chi^2^ = 0.0000	1.000 *
Benzodiazepines	2 (12.50%)	6 (37.50%)	chi^2^ = 2.6667	0.220 *

**Table 2:** Occurrence rate of hospitalizations and visits to the emergency services.

	Hazard Ratio	95% Confidence Interval
Raw Data		
Hospitalizations	0.44	0.17–1.14
Urgent attendances	0.25	0.05–1.16
Controlling for Psychotic Features		
Hospitalizations	0.42	0.18–0.96
Urgent attendances	0.25	0.06–1.13

## Data Availability

Data may be provided upon request.

## Supplementary Materials

The following are available online at https://www.mdpi.com/article/10.3390/ijerph18095019/s1, Table S1: Comparison of the demographic and clinical characteristics between the excluded subjects (N = 7) and the exposed cohort (N = 16).
