# Supplementary material for: A Real-World Study of the Association between a Brief Group Psychoeducation and the Course of Bipolar Disorder
Source: Int J Environ Res Public Health. 2021 May 10;18(9):5019. doi: 10.3390/ijerph18095019 (PMC8126006; doi:10.3390/ijerph18095019)
Supplement: Supplementary file 1 [file ijerph-18-05019-s001.zip › ijerph-1179801-supplementary.pdf]

Table S1. Comparison of the demographic and clinical characteristics between the excluded subjects (N = 7) and the exposed cohort (N = 16). *: Fisher's exact test.

	**Exposed Cohort**	**Excluded Subjects**	**Statistical Test**	***p*-Value**
Age [mean (SD)]	37.19 (6.91)	35.07 (7.23)	t = -0.6670	0.5120
Sex, women [N (%)]	9 (56.25%)	3 (42.86%)	chi2 = 0.3500	0.667*
Duration of illness, years [M (SD)]	8.31 (8.87)	8.71 (7.20)	t = 0.1052	0.9172
Educational level [N (%)]			chi2 = 0.0587	1.000*
Primary studies	0 (0%)	0 (0%)		
Secondary studies	6 (37.50%)	3 (42.86%)		
Superior studies	10 (62.50%)	4 (57.14%)		
Basal insight level [N (%)]			chi2 = 2.3896	0.445*
Poor	1 (6.25%)	0 (0.00%)		
Partial	6 (37.50%)	5 (71.43%)		
Good	9 (56.25%)	2 (28.57%)		
Basal treatment adherence level [N (%)]			chi2 = 1.6066	0.596*
Poor	2 (12.50%)	0 (0.00%)		
Partial	2 (12.50%)	2 (28.57%)		
Good	12 (75.00%)	5 (71.43%)		
